# Ultrasound Imaging and Antithrombotic Effects of PLA-Combined Fe_3_O_4_-GO-ASA Multifunctional Nanobubbles

**DOI:** 10.3389/fmed.2021.576422

**Published:** 2021-05-04

**Authors:** Jie Zhang, Zheng Liu, Cunyi Chang, Ming Hu, Yang Teng, Jinjing Li, Xiangyu Zhang, Yanxia Chi

**Affiliations:** ^1^School of Pharmacy, Jiamusi University, Jiamusi, China; ^2^School of Material Science and Engineering, Jiamusi University, Jiamusi, China; ^3^School of Stomatology, Jiamusi University, Jiamusi, China

**Keywords:** graphene oxide, multifunctional nanobubbles, magnetic target, thrombosis, nanobubbles

## Abstract

PLA-combined ferroferric oxide–graphene oxide–aspirin (Fe_3_O_4_-GO-ASA) multifunctional nanobubbles were prepared using the double emulsion-solvent evaporation method. The obtained composite nanobubbles had a regular spherical shape, Zeta potential of (−36.5 ± 10.0) mV, and particle size distribution range of 200–700 nm. The experimental results showed that PLA-combined Fe_3_O_4_-GO-ASA nanobubbles could effectively improve the antithrombin parameters of PT, TT, APTT, and INR, and significantly inhibit thrombosis when the composite nanobubbles with a concentration of 80 mg·mL^−1^ interacted with the rabbit blood. The prepared composite nanobubbles could reach a significant ultrasonic imaging effect and good magnetic targeting under the magnetic field when the nanobubbles' concentration was only 60 mg·mL^−1^.

## Introduction

At present, ultrasound contrast agents with the functions of targeted diagnosis, interventional therapy, and molecular imaging have become a popular research focus (1). The multifunctional ultrasonic contrast agent nanobubbles can not only improve the contrast and clarity of ultrasound images, but can also have other auxiliary effects by combining with other biomedical nanomaterials such as magnetic nanoparticles, drugs, and genes (2–4).

Timely diagnosis and treatment of thrombotic diseases are important for the prognosis and outcome of treatment. At present, the commonly targeted thrombolytic nanoparticles can be roughly divided into three types (5): magnetic nanoparticles loaded with the thrombolytic drugs (6, 7), a polylactic acid polymer material with the thrombolytic drugs (8, 9) and liposomes with thrombolytic drugs (10, 11). Ultrasonic imaging, as a medical diagnostic technology, has advantages such as being non-invasive, having low adverse reactions, and no radiation, as well as a convenient operation and real-time imaging, which means it shows great potential in the diagnosis of thrombotic diseases (12, 13). Ultrasound imaging can not only be used to detect thrombosis symptoms, but also to dissolve blood clots (14). Studies have shown that ultrasound imaging can effectively dissolve the thrombus of the coronary arteries, cerebral aorta, and peripheral arteries, which results from the mechanical and cavitation effect during the ultrasound (15, 16). Platelet is the central link in thrombosis and plays a key role in thrombosis, particularly in arterial and microvascular thrombosis (17, 18). Aspirin (ASA) is one of the most widely used antiplatelet drugs and is commonly used for the primary and secondary prevention and treatment of thrombocytosis or thrombosis.

Graphene oxide (GO) has good water solubility and biocompatibility (19, 20) due to the plentiful oxygen functional groups (such as -OH and -COOH) on its surface. GO can also load drugs with the benzene ring structure through the π-π conjugation effect. These properties have made GO the focus in a wide range of research, such as in biosensor, bone material, targeted deliver medication, and other biomedical fields (21). As a kind of magnetic substance, nano-iron tetroxide (Fe_3_O_4_) particle has various applications in magnetic targeting delivery systems. This is due to the fact that Fe_3_O_4_ particle is non-toxic to cells, has good biocompatibility and excellent magnetic properties, and can be excreted from the body through degradation (22). Magnetic GO is also used in many applications; GO modified with Fe_3_O_4_ nanoparticles was used for the nanocarrier of doxorubicin, which increased the targeted release effect of drugs (23). The magnetic GO functionalized with PEG could be used for localized photothermal ablation *in vitro* for cancer cells (24). In addition, magnetic GO is widely used for magnetic resonance imaging, molecular imaging probing, and other tasks.

In this study, GO was used as the carrier. Fe_3_O_4_ nanoparticles as the magnetic targeting factor grew on its surface *in-situ*, and the GO-Fe_3_O_4_ magnetic targeting complex was obtained. Then acetyl salicylic acid (ASA) was loaded on the surface of GO-Fe_3_O_4_ complex by π-π function. A PLA complexed with Fe_3_O_4_-GO-ASA multifunctional nanobubble was designed and prepared, and this composite nanobubble could perform magnetically targeted ultrasonic imaging on the thrombus site and simultaneously inhibit the thrombosis formation caused by platelet aggregation. This study hoped to provide a new avenue for the research and development of an ultrasonic contrast agent in the thrombosis field.

## Materials and Methods

### Materials

FeCl_2_ and FeCl_3_ was obtained from Beichen Founder Reagent Factory, Tianjin, China. Acetylsalicylic acid was obtained from ASA, McLean Co., Ltd., Shanghai, China and polylactic acid was supplied by PLA, Kemio Chemical Reagent Co., Ltd., Tianjin, China. Polyethylene glycol (PEG), polyvinyl alcohol (PVA), methylene chloride, isopropanol, and anhydrous ethanol were obtained from Kaitong Chemical Reagent Co., Ltd., Tianjin, China, while New Zealand white rabbits and Kunming mice were provided by Jiamusi University Animal Experimental Center, Heilongjiang, China.

### Characterization

The average particle size and Zeta potential of the nanobubbles were measured by laser particle size tester (DLS). The loading rate of ASA in Fe_3_O_4_-GO-ASA complex was determined by UV-visible spectrophotometer (UV-vis), and the chemical composition of GO-ASA complex was determined by Fourier transform infrared spectroscopy (FT-IR). Scanning electron microscope (SEM) and Transmission electron microscopy (TEM) were used to observe the microscopic morphology of PLA-combined Fe_3_O_4_-GO-ASA nanobubbles. The magnetic properties of Fe_3_O_4_ nanoparticles, Fe_3_O_4_-GO complex, and PLA-combined Fe_3_O_4_-GO-ASA nanobubbles were determined by sample vibration magnetometer (VSM).

### Preparation of Fe_3_O_4_-GO Complex

First, GO was prepared by an improved Hummers method (25). Fe_3_O_4_-GO complex was synthesized by *in-situ* growth method. FeCl_2_ of 0.23g and FeCl_3_ of 0.47g were dissolved in the dual-distilled water and placed in a round-bottomed flask. Polyethylene glycol (PEG, M_w_=1,000) was added under stirring at 60°C. GO of 0.15g was dispersed in dual-distilled water and added into the above system. Meanwhile, the pH of this system was kept at 10–11 by the drop wise addition of NaOH solution and the reaction was maintained for 20 min. Then, the system was heated to 80°C and aged for 30 min. The resulting precipitation was repeatedly washed with the dual-distilled water to neutral, and Fe_3_O_4_-GO complex was obtained by vacuum freeze drying.

### Preparation of Fe_3_O_4_-GO-ASA Complex

Of the Fe_3_O_4_-GO complex, 0.1 g was added to an appropriate amount of anhydrous ethanol for ultrasonic dispersion. Then ASA of 0.2 g was added into the dispersion system of Fe_3_O_4_-GO complex and this mixture system was stirred at 25°C for 2 h. Finally, the precipitation was repeatedly washed by the anhydrous ethanol and treated by vacuum freeze drying to obtain the Fe_3_O_4_-GO-ASA complex.

### Preparation of PLA-Combined Fe_3_O_4_-GO-ASA Nanobubbles

PLA-combined Fe_3_O_4_-GO-ASA nanobubbles were prepared by the double emulsion-solvent evaporation method. PLA of 0.6 g was dissolved in the methylene dichloride and a certain amount of the dispersion liquid of Fe_3_O_4_-GO-ASA complex was added, with ultrasonic emulsification under nitrogen for 5 min. The primary emulsion of W/O was obtained this way. Then primary emulsion of W/O was added into the aqueous solution of polyvinylalcohol (PVA) and ultrasonic emulsification was again performed to get the multiple emulsion of W/O/W. Multiple emulsion of W/O/W was added into aqueous solution of isopropyl alcohol. The mixed system was stirred for 6 h and washed with the centrifugal machine three times, and the obtained supernatant was retained for later use. The obtained precipitation was freeze-dried to obtain PLA-combined Fe_3_O_4_-GO-ASA nanobubbles.

### Ultrasonic Imaging Experiment

Of the four New Zealand rabbits, one was injected with 2 mL physiological saline, and the other three were, respectively, injected with 2 mL PLA-combined Fe_3_O_4_-GO-ASA nanobubbles with concentrations of 20, 60, and 100 mg·mL^−1^ physiological saline solution through the auricular vein. The abdominal cavity was opened at the lower abdomen cut of the rabbit xiphoid and the abdominal aorta shell was stripped off. After the abdominal aorta was exposed, this segment of blood vessel was applied with the filter paper that was soaked in FeCl_3_ solution. After 15 min, the filter paper was removed and the abdomen was stitched. The composite nanobubbles suspension liquid was injected through the rabbit ear vein and the ultrasonic test (3.5 MHz) on the abdominal aorta was carried out with and without external magnetic field. This present study received protocol approval from the university's Institutional Animal Care and Use Committee (IACUC).

### Anti-thrombotic Experiment *in vitro*

A certain amount of PLA-combined Fe_3_O_4_-GO-ASA nanobubbles were ultrasonically broken in normal saline for 2 h. The broken solution was, respectively, diluted to 20, 40, 60, 80, and 100 mg·mL^−1^. Some blood was collected from the ear vein of rabbit. 0.1 mL broken solution with the different concentrations interacted with 2 mL of rabbit blood. Then, the obtained blood sample was added into a small test tube containing No.1 surgical line, and the test tube was kept in a water bath of 37°C for 1.5 h. Finally, the formed blood clot was taken, and the wet weight and dry weight were weighed and compared (26).

Another broken solution with different concentrations interacted with 2 mL of rabbit blood for 1 h, and PT, TT, APTT, and INR values of blood samples were, respectively, measured by semi-automatic blood coagulation analyzer (URJT-600, Uritest, Guilin, China).

### Biocompatibility

Kunming mice of 23~25 g were divided into five groups, with three mice in each group. Mice were prostrated and fixed on the experimental table. 0.2 mL of PLA-combined Fe_3_O_4_-GO-ASA nanobubbles (150 mg· mL^−1^) were subcutaneously injected into the back of mice. The control group was injected with 0.2 mL of normal saline. Mice in the control group were sacrificed at 1 day and those of the composite nanobubbles group were, respectively, sacrificed at 1, 3, 9, and 21 days after injection, and the local tissue of the injection site was observed. At the same time, the muscle at the injection site was taken and fixed in 10%formalin solution for preservation. The samples were conventionally dehydrated and embedded with the paraffin. After HE staining, samples were observed with the light microscope (27).

## Results

**Figure 1:**
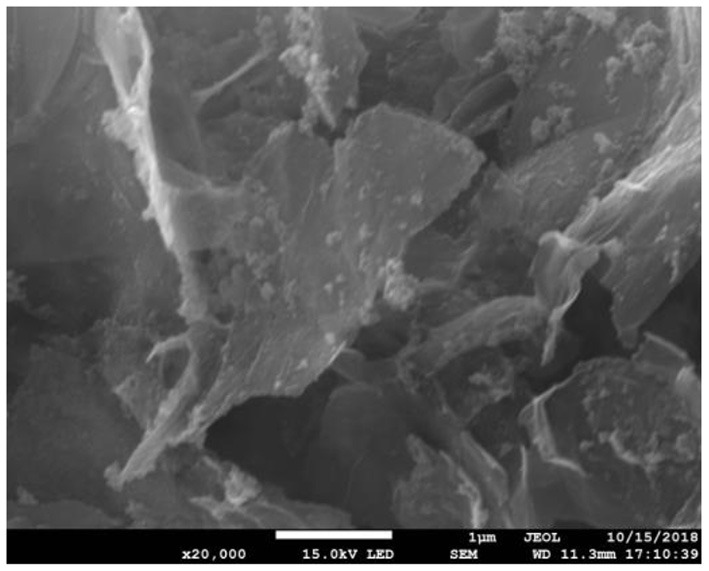
SEM of Fe_3_O_4_-GO complex.

**Figure 2:**
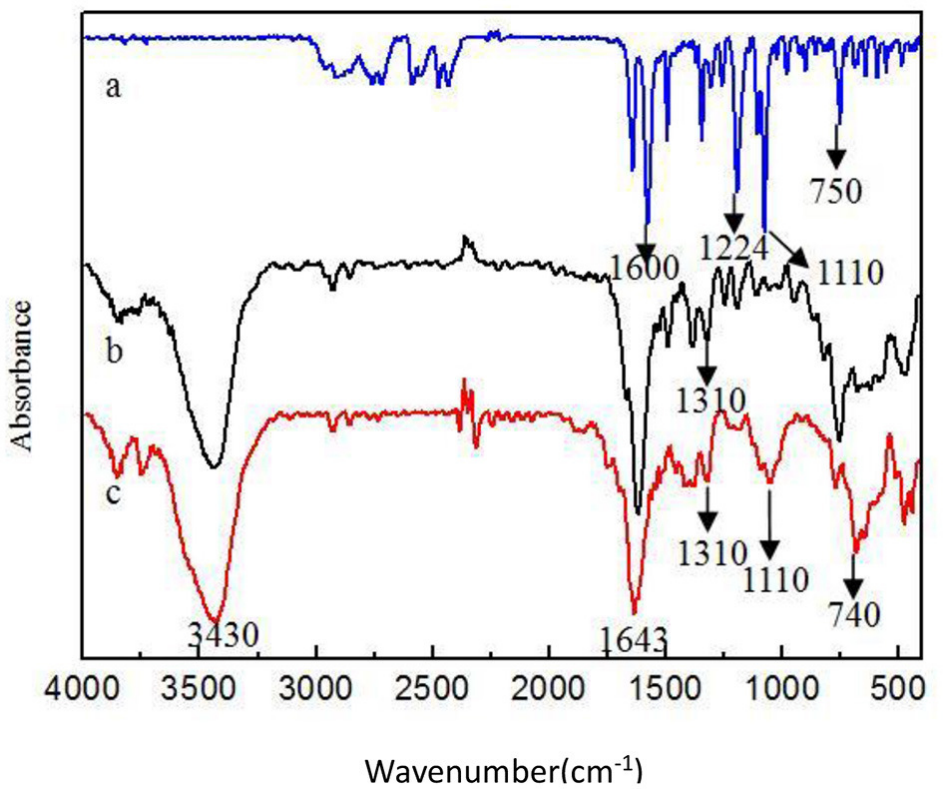
Infrared spectra of different samples **(a)** ASA **(b)** GO **(c)** GO-ASA.

Figure 1 shows SEM of Fe_3_O_4_-GO complex. It can be seen from Figure 1 that there were some nanoparticles on GO nanosheet, which was due to the deposition of Fe_3_O_4_ nanoparticles on the GO by the *in-situ* growth method. Figure 2 shows the infrared spectra of ASA, GO, and GO-ASA complex. In Figure 2a, the absorption peak near 3,000 cm^−1^ was the -COOH characteristic peak, the absorption peak near 1,600 cm^−1^ was the stretching vibration peak of benzene ring skeleton, the absorption peaks at 1,224 and 1,110 cm^−1^ were caused by C-O stretching vibration, and the absorption peak at 750 cm^−1^ was attributed to the vicinal substitution of benzene ring. These peaks were the most obvious characteristics of aspirin. It can be seen from Figure 2b that the absorption peak near 3,430 cm^−1^ was attributed to the stretching vibration peak of –OH in –COOH, the absorption peak at 1,643 cm^−1^ was caused by the bending vibration of –COOH on the edge of GO, and the absorption peak near 1,310 cm^−1^ was the C-O stretching vibration peak. The presence of these oxygen-containing groups indicated that graphite was oxidized. From Figure 2c, the absorption peak at 3,430, 1,643, and 1,310 cm^−1^ belonged to the GO characteristic peak. The absorption peak at 1,110 cm^−1^ was the stretching vibration peak of C-O in ASA. The absorption peak of benzene ring appeared near 740 cm^−1^ and moved toward the low wave number, which was caused by the formed π-hydrogen bond between ASA and GO (28). As a result, ASA was loaded on GO by π-π conjugation in the study.

**Figure 3:**
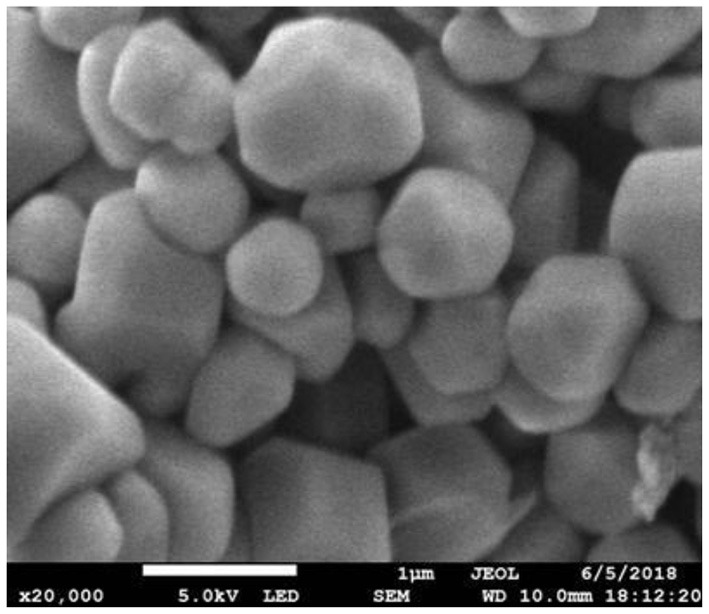
SEM of PLA combined Fe_3_O_4_-GO-ASA nanobubbles.

**Figure 4:**
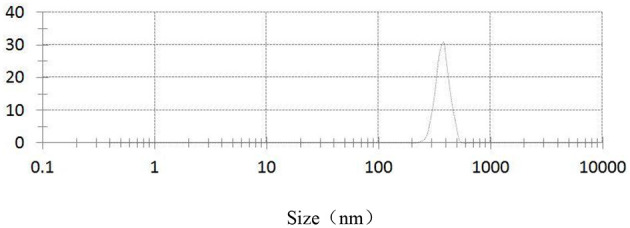
Particle size distribution of PLA combined Fe_3_O_4_-GO-ASA nanobubbles.

**Figure 5:**
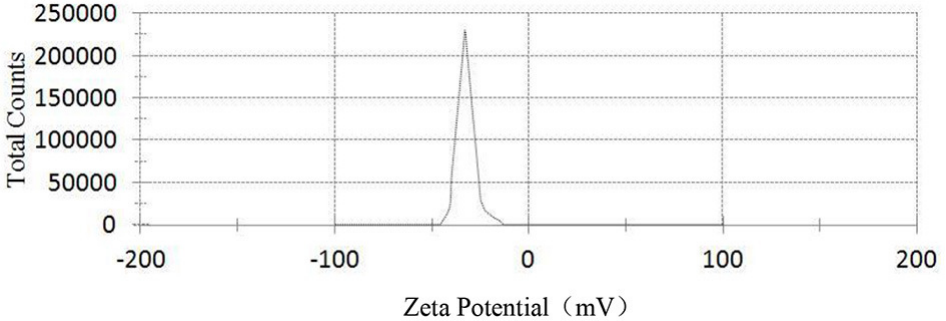
Zeta potential of PLA combined Fe_3_O_4_-GO-ASA nanobubbles.

Figures 3–5, respectively, show the SEM, particle size distribution, and Zeta potential of PLA-combined Fe_3_O_4_-GO-ASA nanobubbles. It can be seen from Figures 3, 4 that the obtained composite nanobubbles had a smooth surface, clear boundary, and uniform size, and the particle size of the nanobubbles was 200–700 nm, which met the clinical requirements of ultrasound contrast agent. Among them, the composite nanobubbles presented the irregular spheres shown in Figure 3, resulting from the freeze drying process of the composite nanobubbles. According to Figure 5, Zeta potential of the composite nanobubbles was (−36.5 ± 10.0) mV, which proved that aqueous solution of the composite nanobubbles had good dispersion stability.

**Figure 6:**
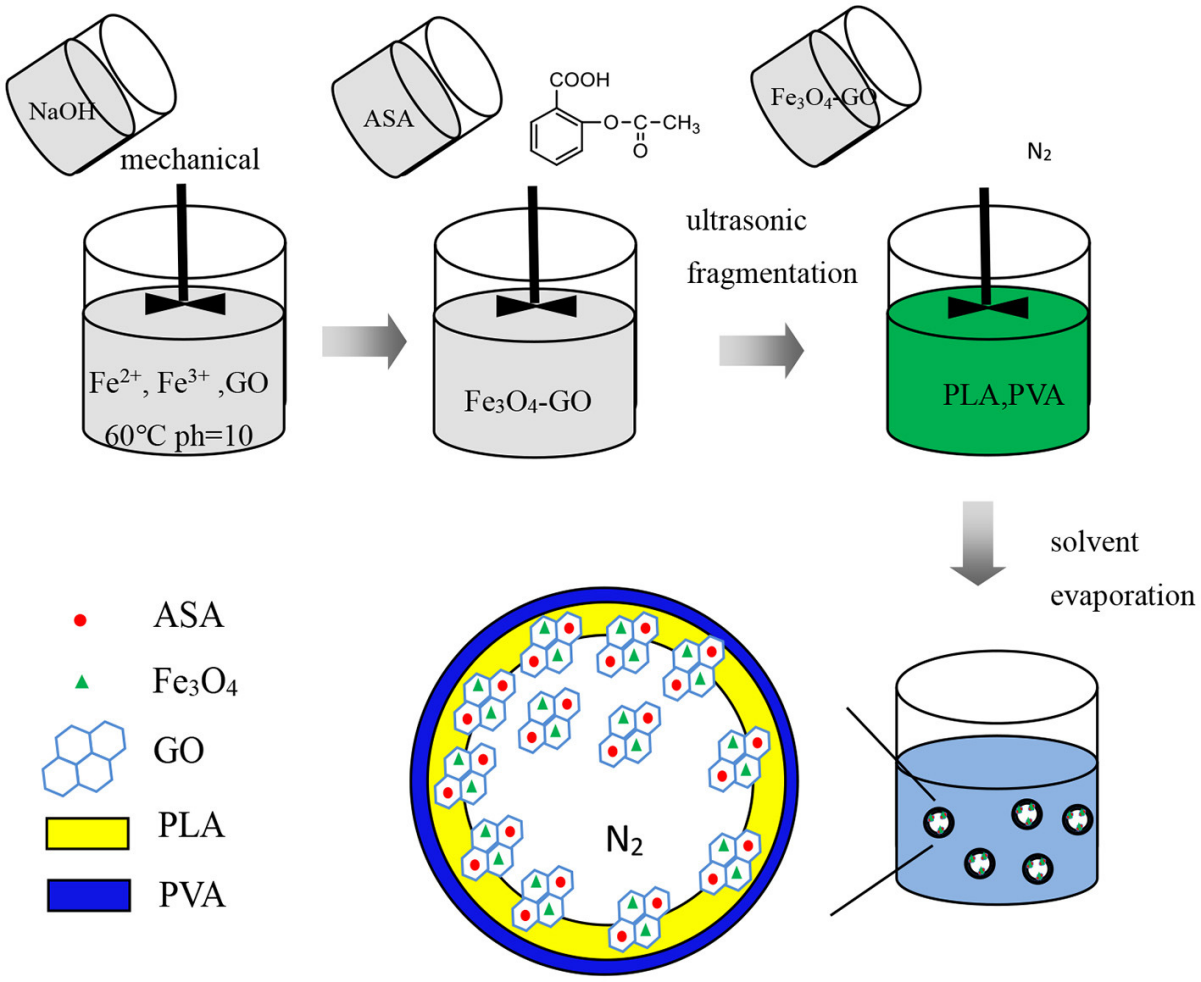
Preparation process of PLA combined Fe_3_O_4_-GO-ASA nanobubbles.

**Figure 7:**
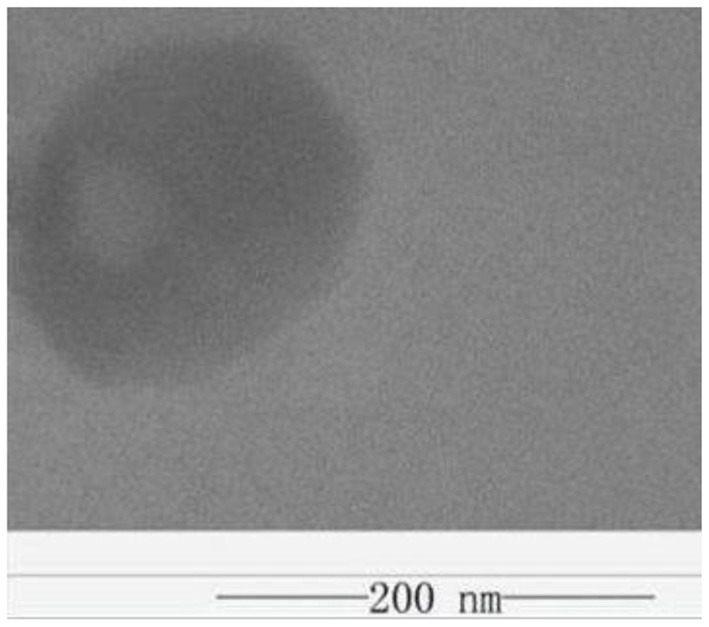
TEM of PLA combined Fe_3_O_4_-GO-ASA nanobubbles.

Figures 6, 7, respectively, show the preparation process and TEM of PLA-combined Fe_3_O_4_-GO-ASA nanobubbles. In the preparation process, Fe_3_O_4_ was firstly grown on the GO surface by an *in-situ* growth method and ASA was loaded on Fe_3_O_4_-GO by π-π conjugation effect to obtain Fe_3_O_4_-GO-ASA complex. Then, Fe_3_O_4_-GO-ASA complex was encapsulated in PLA nanobubbles by double emulsion-solvent evaporation method. During the first ultrasonic emulsification, PLA solution was destroyed into some small droplet wrapping Fe_3_O_4_-GO-ASA complex in the inner water phase. Due to the continuous N_2_ that was imported in the process of ultrasonic breaking, small bubbles also contained N_2_. At this moment, the obtained W/O phase was transferred to the PVA solution and evenly stirred, and the second ultrasonic emulsification was performed. As a result, PVA would coat on the surface of PLA nanobubbles by hydrogen bonding to form a hard shell (29). After the vacuum freeze drying, the water in the nanobubbles disappeared, and Fe_3_O_4_-GO-ASA complex would gather at the inner side of the nanobubble or embed in the film of the nanobubble due to the surface tension. As shown in Figure 7, the film and inner side of the nanobubble presented a black shadow.

**Figure 8:**
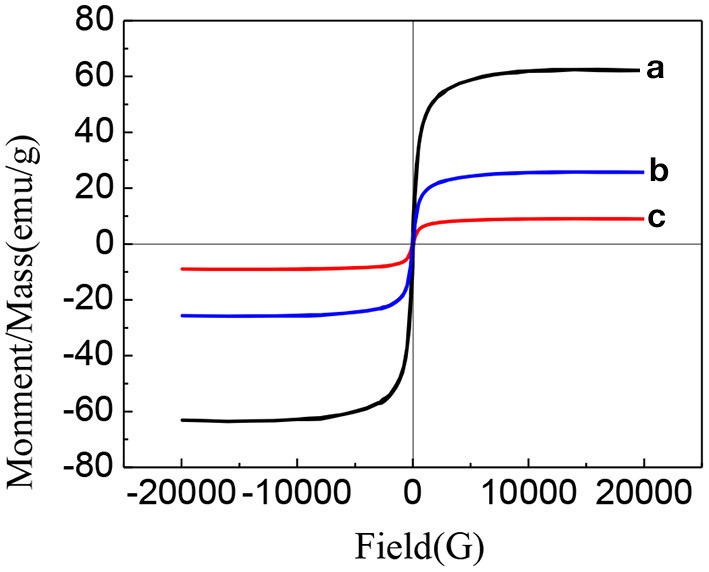
Hysteresis curves of different samples **(a)** Fe_3_O_4_ **(b)** Fe_3_O_4_-GO complex **(c)** PLA combined Fe_3_O_4_-GO-ASA nanobubbles (30, 31).

Figure 8 shows the hysteresis curves of Fe_3_O_4_, Fe_3_O_4_-GO complex, and PLA-combined Fe_3_O_4_-GO-ASA nanobubbles (30, 31). As can be seen from Figure 8, the hysteresis curves of Fe_3_O_4_, Fe_3_O_4_-GO complex, and PLA-combined Fe_3_O_4_-GO-ASA nanobubbles presented a similar pattern, meaning that their magnetic behaviors were similar. There was no coercive field nor residual magnetism at the zero point, which indicated that three samples had paramagnetic characteristics and could gather under external magnetic field conditions (32). In addition, the specific saturation magnetization intensity of Fe_3_O_4_, Fe_3_O_4_-GO complex and PLA-combined Fe_3_O_4_-GO-ASA nanobubbles decreased orderly; this was because the Fe_3_O_4_ content in the three samples of the same mass decreased in sequence, indicating that the magnetism of the Fe_3_O_4_-GO complex and PLA-combined Fe_3_O_4_-GO-ASA nanobubbles were derived from Fe_3_O_4_.

**Figure 9:**
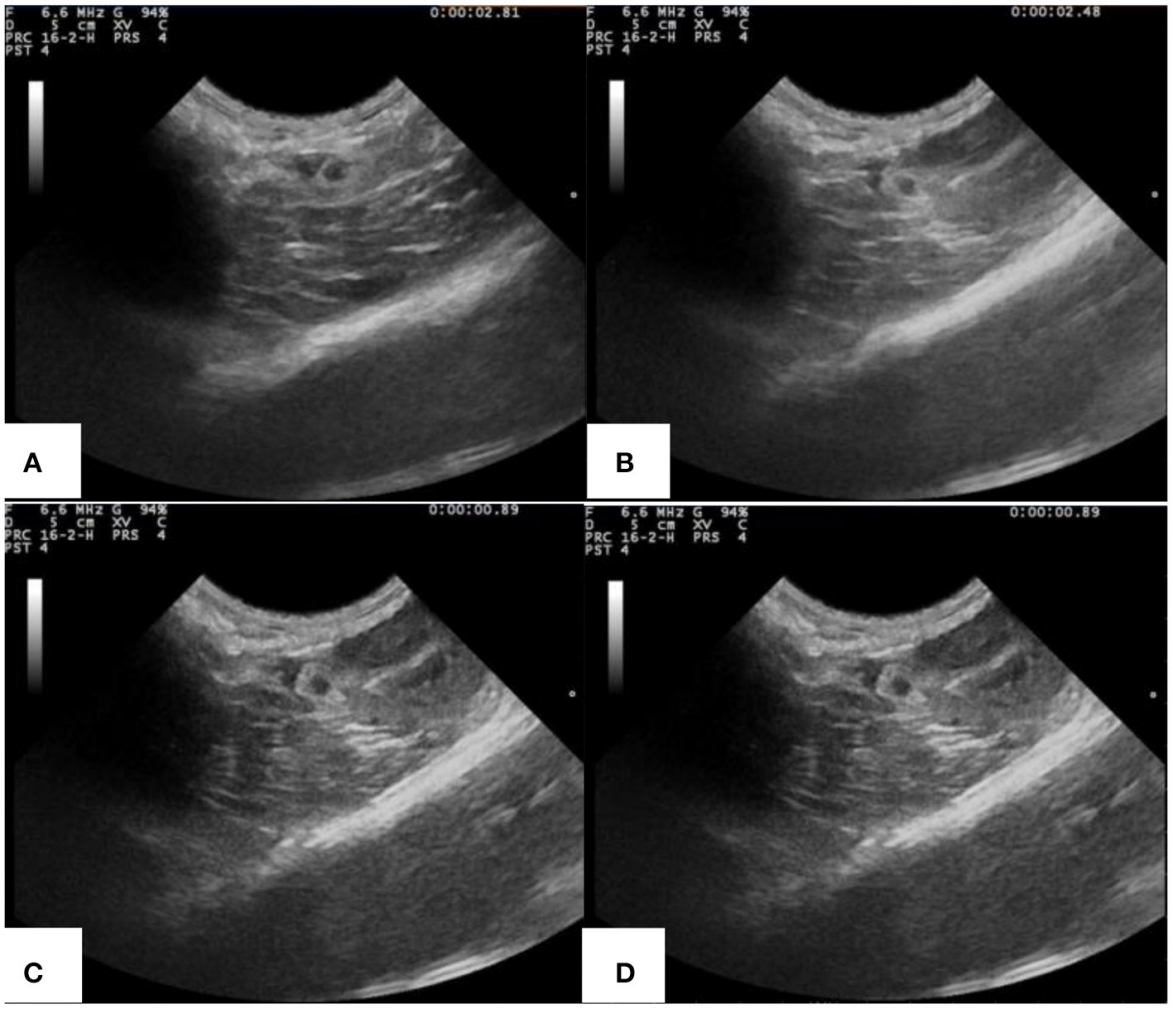
Ultrasonic imaging of rabbit abdominal aorta of rabbits injected with PLA combined Fe_3_O_4_-GO-ASA nanobubbles of the different concentrations **(A)** 0 mg·mL^−1^ **(B)** 20 mg·mL^−1^ **(C)** 60 mg·mL^−1^ **(D)** 100 mg·mL^−1^.

Figure 9 is the ultrasonic imaging of rabbit abdominal aorta of PLA-combined Fe_3_O_4_-GO-ASA nanobubbles with different concentrations. As can be seen from Figure 9A, when PLA-combined Fe_3_O_4_-GO-ASA nanobubbles were not injected, the ultrasonic imaging of rabbit abdominal aorta was dark and very fuzzy. When the concentration of PLA-combined Fe_3_O_4_-GO-ASA nanobubbles were 20 mg·mL^−1^ (Figure 9B), the ultrasonic signal was enhanced, and the contrast and identification of the image was significantly improved. When the concentration of PLA-combined Fe_3_O_4_-GO-ASA nanobubbles was >60 mg·mL^−1^ (Figures 9C,D), the effect of ultrasound imaging for the abdominal aorta was significantly improved, while the contrast of the image did not significantly change with the increase of PLA-combined Fe_3_O_4_-GO-ASA nanobubbles concentration. As a result, the ultrasonic effect had reached the requirements of ultrasound imaging when the concentration of PLA-combined Fe_3_O_4_-GO-ASA nanobubbles was 60 mg·mL^−1^.

**Figure 10:**
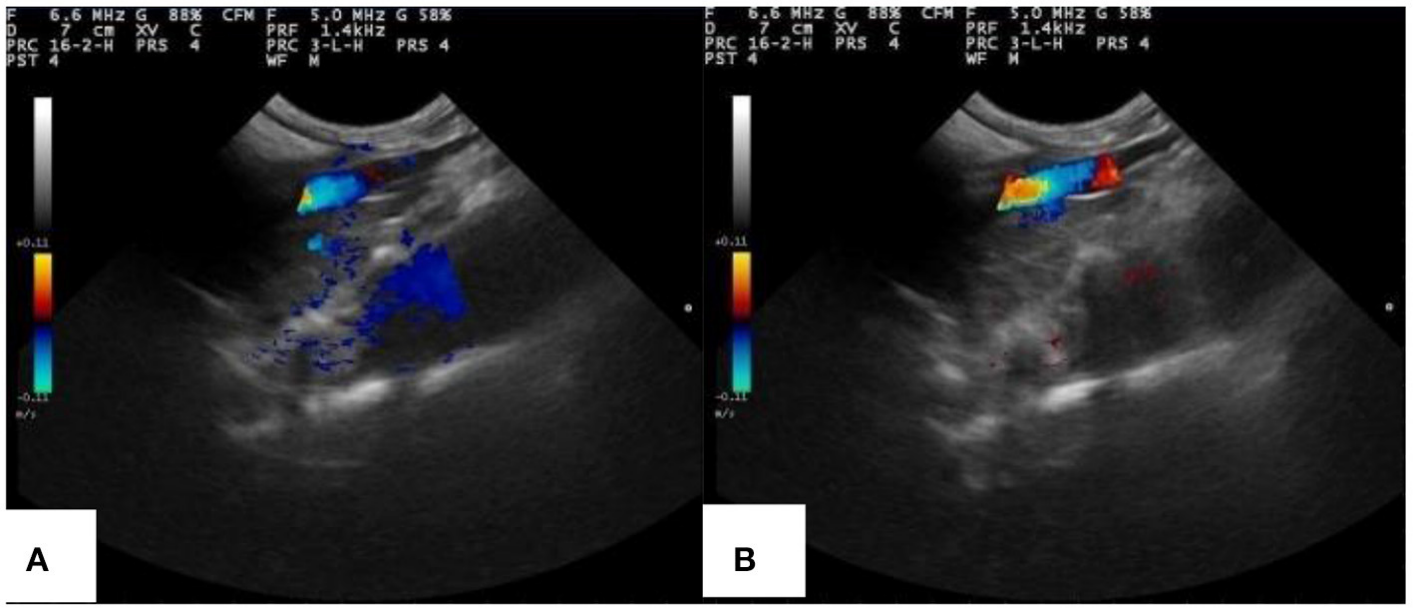
Ultrasonic imaging of rabbit abdominal aorta of rabbits injected with PLA combined Fe_3_O_4_-GO-ASA nanobubbles **(A)** without the magnetic field **(B)** with the additional magnetic field in the thrombus area.

PLA-combined Fe_3_O_4_-GO-ASA nanobubbles were injected into the model rabbit with abdominal aortic thrombus, and ultrasonic imaging photos of rabbit abdominal aorta with and without magnetic field are shown in Figure 10. From Figure 10A, a strong and distinct blue signal appeared in the right side of the thrombus, indicating the reflux blood presented at the thrombus obstruction site. At the same time, the composite nanobubbles were carried by blood and would flow throughout the body, and the ultrasonic signals could present in the tissues or organs where the composite nanobubbles passed through. As a result, a weak and large dark blue signal also appeared below the abdominal aorta of the rabbit. It can be seen from Figure 10B that when the magnetic field was added in the abdominal aorta region, the intensity of ultrasonic signal in this region was significantly enhanced, while the signal intensity in other regions was significantly weakened, indicating that PLA-combined Fe_3_O_4_-GO-ASA nanobubbles could effectively enrich in the magnetic field region. Thus, the obtained PLA-combined Fe_3_O_4_-GO-ASA nanobubbles had a magnetic targeting effect under the action of an external magnetic field, which could significantly enhance the image clarity of the observed site without increasing the dosage of the composite nanobubbles.

Table 1 shows the wet weight and dry weight of the obtained coagulated blood clot due to the interaction between PLA-combined Fe_3_O_4_-GO-ASA nanobubbles with different concentrations and blood samples. It can be seen from Table 1 that the wet weight of the normal saline control group was 95.10 ± 0.22 mg, while the wet weights for the composite nanobubbles with the concentrations of 20, 40, 60, 80, and 100 mg·mL^−1^ were, respectively, 87.27 ± 0.16 mg, 75.73 ± 0.47 mg, 56.57 ± 0.32 mg, 44.80 ± 0.16 mg, and 36.03 ± 0.22 mg, showing the composite nanobubbles were very significantly different from the control group (*P* < 0.01). The dry weight of the normal saline control group was 52.03 ± 0.35 mg and the dry weight of the composite nanobubble with the concentration of 20 mg·mL^−1^ was 50.17 ± 0.35 mg; it was significantly different from the control group (0.01 < P < 0.05). When the composite nanobubble concentration was 40, 60, 80, and 100 mg·mL^−1^, their dry weights were, respectively, 44.83 ± 0.36 mg, 36.67 ± 0.50 mg, 33.03 ± 0.75 mg, and 27.57 ± 0.60 mg and had very significant differences in comparison with the control group (*P* < 0.01). We could see that the wet weight and dry weight of clots significantly decreased with the concentration increase of the composite nanobubbles.

**Table 1:** Wet weight and dry weight of the coagulated blood clot.

**Concentration (mg·mL^**−1**^)**	**Wet weight (mg)**	**Dry weight (mg)**
0	95.10 ± 0.22	52.03 ± 0.35
20	87.27 ± 0.16^**^	50.17 ± 0.35^*^
40	75.73 ± 0.47^**^	44.83 ± 0.36^**^
60	56.57 ± 0.32^**^	36.67 ± 0.50^**^
80	44.80 ± 0.16^**^	33.03 ± 0.75^**^
100	36.03 ± 0.22^**^	27.57 ± 0.60^**^

* *P < 0.05,*

** *P < 0.01*.

This was because the ASA content in the composite nanobubbles increased with the increase of the composite nanobubbles concentration, and ASA had an inhibitory effect on the thrombosis (33). The antithrombotic effect of ASA is mainly realized by inhibiting the activity of the platelet cyclooxygenase and preventing the synthesis and release of prostaglandins and thromboxane (34). Moreover, we could conclude from Table 1 that the ratio of wet weight and dry weight of clots decreased with the increase of the composite nanobubbles concentration. For the formation of thrombus, the ratio of wet weight and dry weight ratio is smaller and the dissociation of thrombus factor in the blood is stronger, thus the speed of thrombosis caused by factors becomes slow. The ratio of wet weight and dry weight is lower, indicating that the risk of thrombosis is smaller. It can be seen that the anti-thrombotic ability of PLA-combined Fe_3_O_4_-GO-ASA nanobubbles increased with the increase of its concentration.

Table 2 shows the changes of the coagulated blood parameters due to the interaction between PLA-combined Fe_3_O_4_-GO-ASA nanobubbles of different concentrations and blood samples. As can be seen from Table 2, the values of PT, TT, APTT, and INR in blood samples showed an increasing trend with the increase of the composite nanobubbles concentration. Specifically, APTT value significantly increased when the composite nanobubbles concentration was 20 mg·mL^−1^, PT and TT values significantly increased at the concentration of 40 mg·mL^−1^, and INR value significantly increased at the concentration of 80 mg·mL^−1^. It can be seen that when the concentration of the composite nanobubbles was above 80 mg·mL^−1^, they could effectively promote the synthesis of cAMP in platelet and interdict the platelet membrane glycoproteins receptors, which reduced the concentrations of thromboxane A and phosphodiesterase in plasma and eventually inhibited the formation process of thrombosis. So, PLA-combined Fe_3_O_4_-GO-ASA nanobubbles could effectively inhibit thrombosis and achieve the purpose of adjuvant therapy.

**Table 2:** Effects of PLA combined Fe_3_O_4_-GO-ASA nanobubbles with the different concentrations on the coagulation blood parameters.

**Concentration (mg·mL**^**−1**^**)**	**PT(s)**	**TT(s)**	**APTT(s)**	**INR(s)**
0	13.2	14.0	29.6	1.09
10	13.5	14.5	29.7	1.11
20	13.7	15.7	30.1	1.16
40	15.3	16.4	30.9	1.23
80	17.2	18.0	32.8	1.45
160	18.8	20.1	33.6	1.56

**Figure 11:**
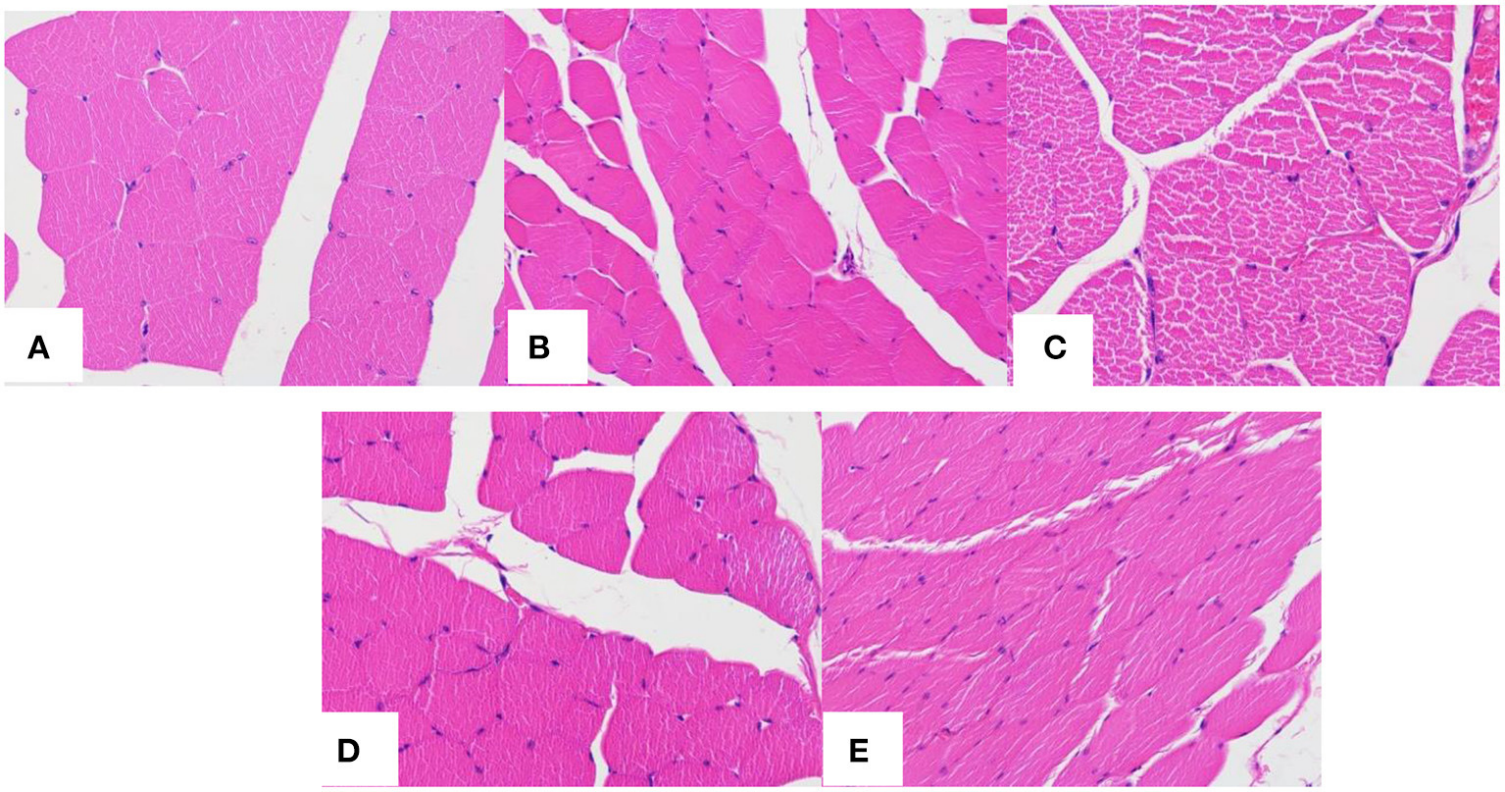
HE staining pictures of mouse muscle after the subcutaneous injection of saline at 1 day **(A)** and the composite nanobubbles at 1 day **(B)**, 3 days **(C)**, 9 days **(D)**, and 21 days **(E)**.

Figure 11 is the HE staining pictures of mouse muscle after the subcutaneous injection of normal saline and PLA-combined Fe_3_O_4_-GO-ASA nanobubbles at different times. As shown in Figure 11A, mouse muscle tissue had an intact structure and the histopathological change was not observed for the normal saline group. For PLA-combined Fe_3_O_4_-GO-ASA nanobubbles, the injection site of 1 day formed a cyst by the experimental observation, while the cyst had a thin wall and it contained the nanobubbles injection solution and interstitial fluid. As shown in Figure 11B, compared with the saline group, there was a slight inflammatory cell infiltration at the injection site. After PLA-combined Fe_3_O_4_-GO-ASA nanobubbles were injected for 3 days, the formed cyst at the injection site significantly reduced, and the inflammatory cell infiltration was weakened as shown in Figure 11C, however, the infiltration phenomenon did not disappear because the degradation products of the composite nanobubbles were not eliminated or absorbed in a timely manner. At 9 days after injection, the local cyst basically disappeared, and the inflammatory cell infiltration significantly reduced (Figure 11D). At 21 days after injection, the inflammatory cell infiltration disappeared (Figure 11E), and there was no obvious change in the deep muscle tissue. Therefore, the injection of PLA-combined Fe_3_O_4_-GO-ASA nanobubbles only caused a slight inflammatory response, and the accumulation of tissue penetrating fluid or obvious proliferation of vascular or fibers was not observed, indicating that PLA-combined Fe_3_O_4_-GO-ASA nanobubbles have good biocompatibility.

## Conclusions

In this study, the nanobubbles were prepared in three steps. Firstly, Fe_3_O_4_ nanoparticles were deposited on GO using an *in-situ* growth method. Then, ASA was adsorbed on the Fe_3_O_4_- GO composite through π-π effect. Finally, the newly formed Fe_3_O_4_-GO-ASA composite was combined into the PLA nanobubbles by the double emulsion-solvent evaporation method.

The obtained PLA-combined Fe_3_O_4_-GO-ASA nanobubbles can inhibit thrombosis and effectively enrich the target area under the action of a magnetic field to achieve the targeted imaging effect. Moreover, if the ultrasonic power was increased, the composite nanobubbles could be broken and the controlled drug release achieved, indicating that PLA-combined Fe_3_O_4_-GO-ASA nanobubbles could realize the dual functions of targeted drug delivery and controlled release. However, the antithrombotic performance of PLA-combined Fe_3_O_4_-GO-ASA nanobubbles *in vivo* will need further study. This study lays a foundation for the development and application of magnetic targeted ultrasound contrast agents.

## Data Availability Statement

The original contributions presented in the study are included in the article/Supplementary Material, further inquiries can be directed to the corresponding author/s.

## Ethics Statement

The animal study was reviewed and approved by Ethics Committee of Jiamusi university Jiamusi university.

## Author Contributions

JZ and YC are responsible for the design of experimental direction, arrangement of experimental methods, revision, and proofreading of this paper. ZL and CC were responsible for the implementation of the experiment and the preliminary writing of the paper. MH, YT, JL, and XZ were responsible for the evaluation of experimental design.

## Conflict of Interest

The authors declare that the research was conducted in the absence of any commercial or financial relationships that could be construed as a potential conflict of interest.
