# Supplementary material for: Ultrasound Imaging and Antithrombotic Effects of PLA-Combined Fe3O4-GO-ASA Multifunctional Nanobubbles
Source: Front Med (Lausanne). 2021 May 4;8:576422. doi: 10.3389/fmed.2021.576422 (PMC8129036; doi:10.3389/fmed.2021.576422)
Supplement: Supplementary file 1 [file Data_Sheet_1.doc]

*Determination of ASA loading rate in Fe_3_O_4_-GO-ASA complex*

ASA standard curve: ASA standard solutions with the concentrations of 0.04 mg·mL^-1^, 0.06 mg·mL^-1^, 0.08 mg·mL^-1^, 0.10 mg·mL^-1^ and 0.12 mg·mL^-1^ were prepared with the absolute ethanol as solvent, respectively. The absorbances of ASA standard solutions were measured by UV-vis at 275 nm and an ASA standard curve was obtained.

Five groups of Fe_3_O_4_-GO-ASA complex, each group of 10 mg, were put into the absolute ethanol and treated for 2 h under the ultrasonic condition. After filtration, the obtained filtrate was been constant in 10 mL and their absorbances were determined at 275 nm. ASA loading rate in Fe_3_O_4_-GO-ASA complex was calculated according to ASA standard curve.

*Sample recovery rate:* Five groups of filtrates of Fe_3_O_4_-GO-ASA complex was obtained by the above method. Then, 1.0 mg, 2.0 mg, 3.0 mg, 4.0 mg, and 5.0 mg of ASA were respectively added into the 5 groups of filtrates and their absorbances were measured at 275 nm, and the ASA recovery rate was calculated.

Fig.1 shows the ASA standard curve and Table 1 is the absorbances of ASA in Fe_3_O_4_-GO-ASA complex filtrate. From Fig.1 and Table 1, the average concentration of ASA in the five filtrates of Fe_3_O_4_-GO-ASA complex was 0.073 mg·mL^-1^, and the ASA content in Fe_3_O_4_-GO-ASA complex was 7.3%. Table 2 is the recovery test results of ASA. Average value of the adding sample recovery of ASA was 97.84±0.52 and RSD % was 0.52%, which indicated that the measurement of ASA content in Fe_3_O_4_-GO-ASA complex had high accuracy and met the relevant requirements of the test.

Table 1 The absorbance of ASA in Fe_3_O_4_-GO-ASA complex filtrate

Table 2 The recovery test results of ASA

Table 1

| Times | 1 | 2 | 3 | 4 | 5 | average value |
| --- | --- | --- | --- | --- | --- | --- |
| Absorbance | 0.281 | 0.273 | 0.304 | 0.269 | 0.296 | 0.285 |

Table 2

| Times | 1 | 2 | 3 | 4 | 5 | average value | RSD % |
| --- | --- | --- | --- | --- | --- | --- | --- |
| Recovery rate | 98.1 | 98.4 | 97.5 | 97.0 | 98.2 | 97.84±0.52 | 0.52 |

Fig.1 Standard curve line of ASA


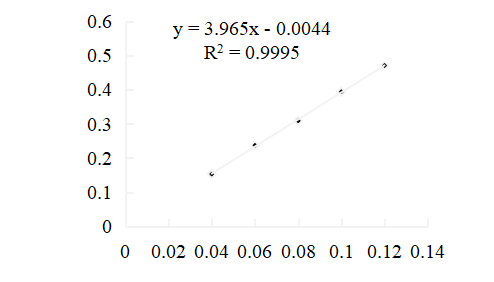


Absorbance

concentration of the ASA(mg·mL^-1^)

(mg·mL^-1^)

Fig. 1
